# A laser guide technique: a novel method for accurate acetabular cup alignment in total hip arthroplasty

**DOI:** 10.1038/s41598-022-21975-x

**Published:** 2022-10-29

**Authors:** Yusuke Kohno, Tetsuro Nakamura, Masanori Fujii, Satoshi Shin, Toshihiko Hara

**Affiliations:** 1 Department of Orthopaedic Surgery, Japan Community Health care Organization (JCHO) Kyushu Hospital, 1-8-1 Kishinoura, Yahatanishi-ku, Kitakyushu, 806-8501 Japan; 2 Department of Orthopaedic Surgery, Iizuka Hospital, 3-83 Yoshiomachi, Iizuka, 820-8505 Japan

**Keywords:** Outcomes research, Orthopaedics, Surgery

## Abstract

For accurate cup alignment without navigation in total hip arthroplasty (THA), we developed a “laser guide technique.” The major purposes of this paper are to introduce the technique and compare its accuracy with a conventional manual technique. As a clinical outcome, the dislocation rate was reviewed. Our laser guide technique, which includes preoperative postural adjustment and intraoperative angular reference, has been detailed in the manuscript. 599 hips in 523 patients who underwent primary THA with piriformis-sparing posterolateral approach in April 2010–March 2016 were reviewed. Patients were divided into three groups: conventional group (135 hips), laser guide group (80 hips), and laser + radiographic alignment guide group (384 hips). Radiographic inclination (RI) and radiographic anteversion (RA) errors were evaluated. The dislocation rate was reviewed in 540 hips in 476 patients who were followed up > 2 years postoperatively. Absolute values of the RI/RA error in the three groups were 5.3° ± 4.0°/6.5° ± 4.5°, 4.0° ± 2.8°/4.9° ± 4.4°, and 3.3° ± 2.6°/3.6° ± 2.8°, respectively, indicating substantially enhanced accuracy with laser and radiographic alignment guide. The dislocation rates were 2.5% (3/119) and 0.2% (1/421) in the conventional and laser groups, respectively. Our novel laser guide technique considerably enhanced cup alignment accuracy, suggesting its potential applicability for THA in the lateral decubitus position.

## Introduction

Suboptimal cup alignment in total hip arthroplasty (THA) is associated with a decreased range of motion and increased instability, which lead to hip dislocation^1^. The dislocation is one of the most common complications after THA^2^ and is a common cause of revision THA^3,4^. The frequency of dislocation after primary THA is estimated to be 1–5%^5–11^. Cup malalignment is also related to increased biomechanical stress, leading to bearing surface wear and osteolysis^12,13^, which affect implant longevity. Three-dimensional preoperative planning and accurate implant placement accordingly are required in modern THA to avoid the above complications and get better clinical outcomes.

To place an acetabular cup as planned preoperatively, CT-based navigation or robotic arm-assisted surgery is certainly beneficial^14–16^. However, currently these systems are far from being widely used for several reasons mainly because of a very high cost. On the other hand, poor accuracy of freehand cup placement has been reported^17,18^. Callanan et al.^5^ reported that only 47% of cups were optimally positioned in both inclination (30°–45°) and anteversion (5°–25°) angles. Particularly in THA with patients in the lateral decubitus position, the variety of pelvic tilt and rotation during surgery is another cause of the cup positioning error^19,20^. To overcome these problems, we have developed a novel laser guide technique, which does not require a costly navigation system. This technique includes a preoperative postural adjustment to the functional pelvic plane (FPP) in the lateral decubitus position and an intraoperative angular reference guide for an acetabular cup using a laser beam.

The major purposes of this study are (1) to introduce our novel laser guide technique for acetabular cup placement in THA in the lateral decubitus position and (2) to compare the accuracy of the laser guide technique and a conventional manual technique. As a clinical outcome of our THA with the piriformis-sparing posterolateral approach, the dislocation rate was reviewed.

## Materials and methods

### Patients

**Figure 1:**
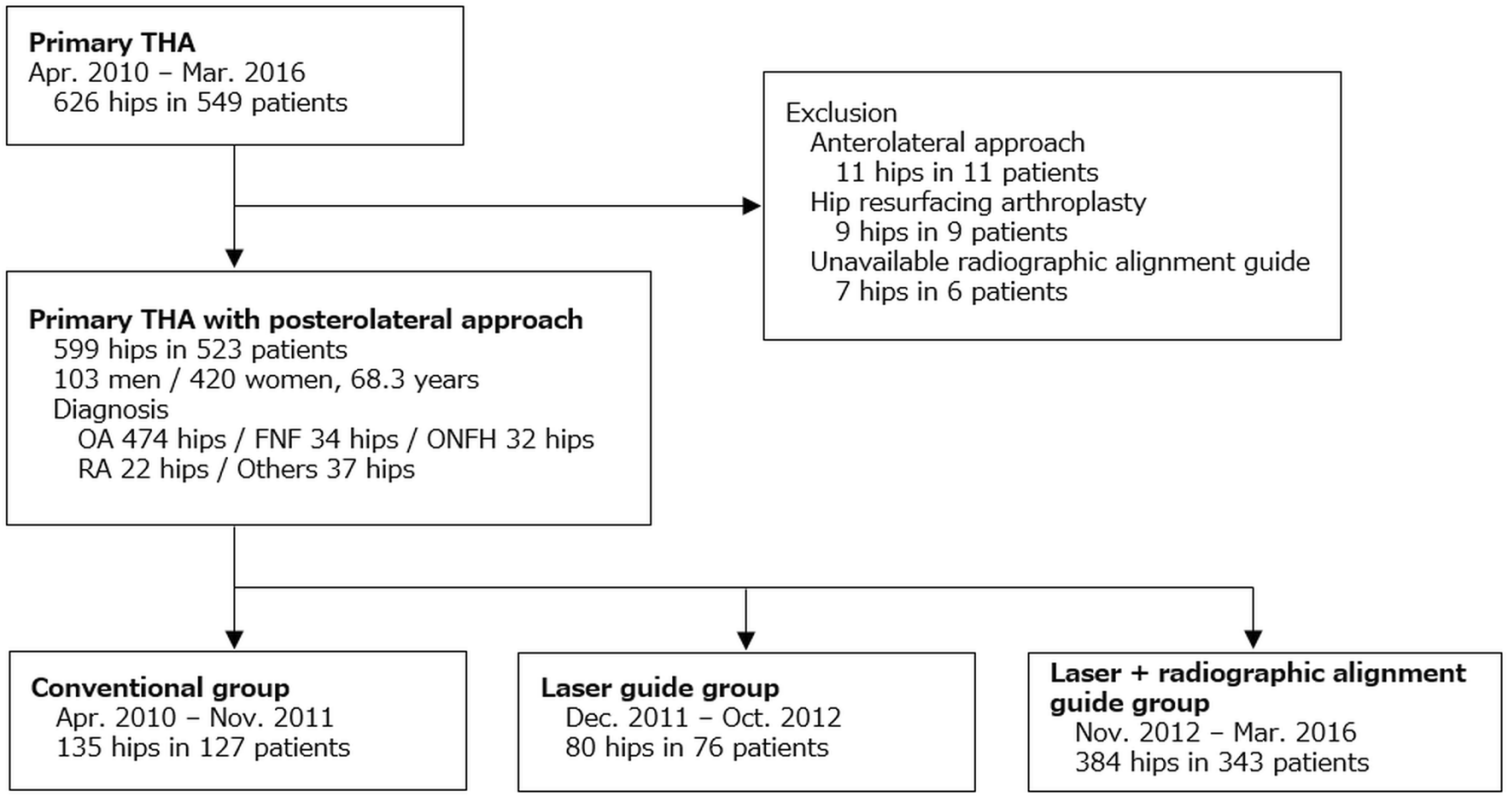
Patients selection. Demographic data and grouping by surgery period. *THA* total hip arthroplasty, *OA* osteoarthritis, *FNF* femoral neck fracture, *ONFH* osteonecrosis of the femoral head, *RA* rheumatoid arthritis.

Our institutional review board approved this retrospective case–control study (Japan Community Health care Organization (JCHO) Kyushu hospital medical ethics board, approval No. 793). It was conducted in accordance with the Declaration of Helsinki. In accordance with the Ethical Guidelines for Medical and Biological Research Involving Human Subjects in Japan, informed consent was waived by the JCHO Kyushu hospital medical ethics board because this study involved the secondary analysis of existing anonymized information in our institution. Between April 2010 and March 2016, among all 626 hips in 549 patients with primary THA at our single institution, 11 hips in 11 patients with anterolateral approach, 9 hips in 9 patients with hip resurfacing arthroplasty, and 7 hips in 6 patients, in whom surgery radiographic alignment guide was unavailable, were excluded. The remaining 599 hips in 523 patients were included in this study. There were 103 men (116 hips) and 420 women (483 hips). The mean patient age at surgery was 68.3 years (range, 37–90 years). The indications for THA were osteoarthritis (474 hips), femoral neck fracture (34 hips), osteonecrosis of the femoral head (32 hips), rheumatoid arthritis (22 hips), and other diseases including rapidly destructive coxarthropathy and subchondral insufficiency fracture (37 hips) (Fig. 1).

The patients were divided into the following three groups depending on the surgery period: (1) conventional group (conventional THA performed between April 2010 and November 2011, 135 hips in 127 patients), (2) laser guide group (THA with laser guide technique between December 2011 and October 2012, 80 hips in 76 patients), and (3) laser + radiographic alignment guide group (THA with laser guide technique using radiographic cup alignment guide between November 2012 and March 2016, 384 hips in 343 patients) (Fig. 1). AMS HA shell (Kyocera Medical, Osaka, Japan) was used until March 2015, and SQRUM HA shell (Kyocera Medical) was used thereafter.

### Preoperative planning

**Figure 2:**
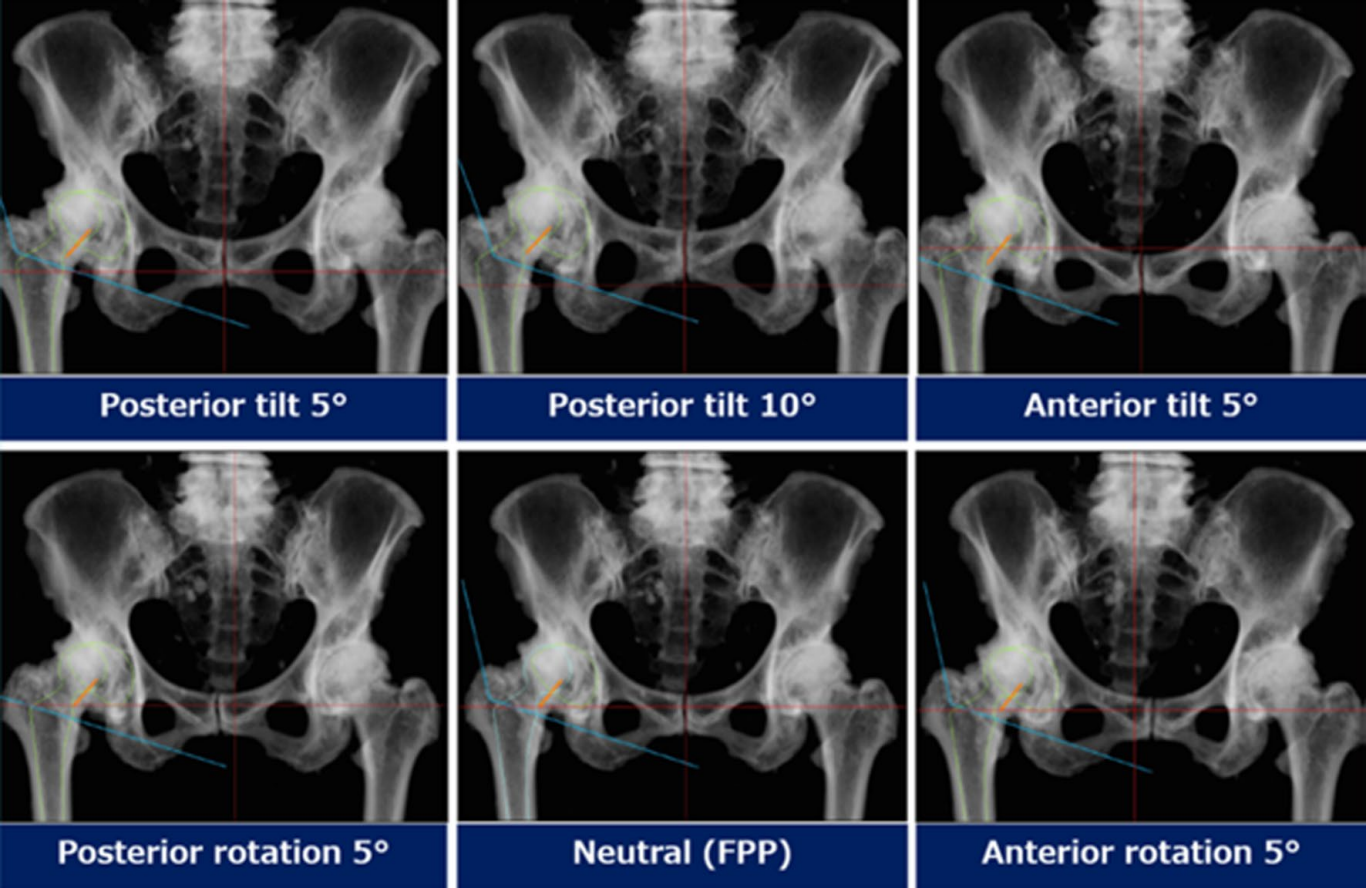
Several patterns of digitally reconstructed radiographs of the pelvis include a pelvis in the functional pelvic plane (FPP) and pelvises that changed pelvic tilt or pelvic rotation from the FPP. The pelvic position is adjusted based on these images just before surgery.

Pelvic CT was performed with the patients in a supine position preoperatively, and the images were obtained at 1-mm intervals from the superior edge of the pelvis to just below the knee joint line^21^. Using the CT data, the implant size and positioning were three-dimensionally planned using 3D Template (Kyocera Medical). Following the pelvic position was corrected to the FPP, cup alignment was planned with 40° (after 2012) or 45° (before 2011) in radiographic inclination (RI) and 20° in radiographic anteversion (RA). Target RA was changed in the range of ± 5° taking acetabular morphology, spinopelvic mobility, and stem anteversion into account. Several patterns of digitally reconstructed radiographs (DRRs) of the pelvis were also prepared, which include a pelvis in the FPP and pelvises that changed pelvic tilt (change in the sagittal plane) or pelvic rotation (change in axial plane) from the FPP (Fig. 2) for the reference of pelvic postural correction before surgery.

### Surgical technique

THA with the piriformis-sparing posterolateral approach^22,23^ was performed in all included patients. Our surgical technique was gradually updated as mentioned below.

**Figure 3:**
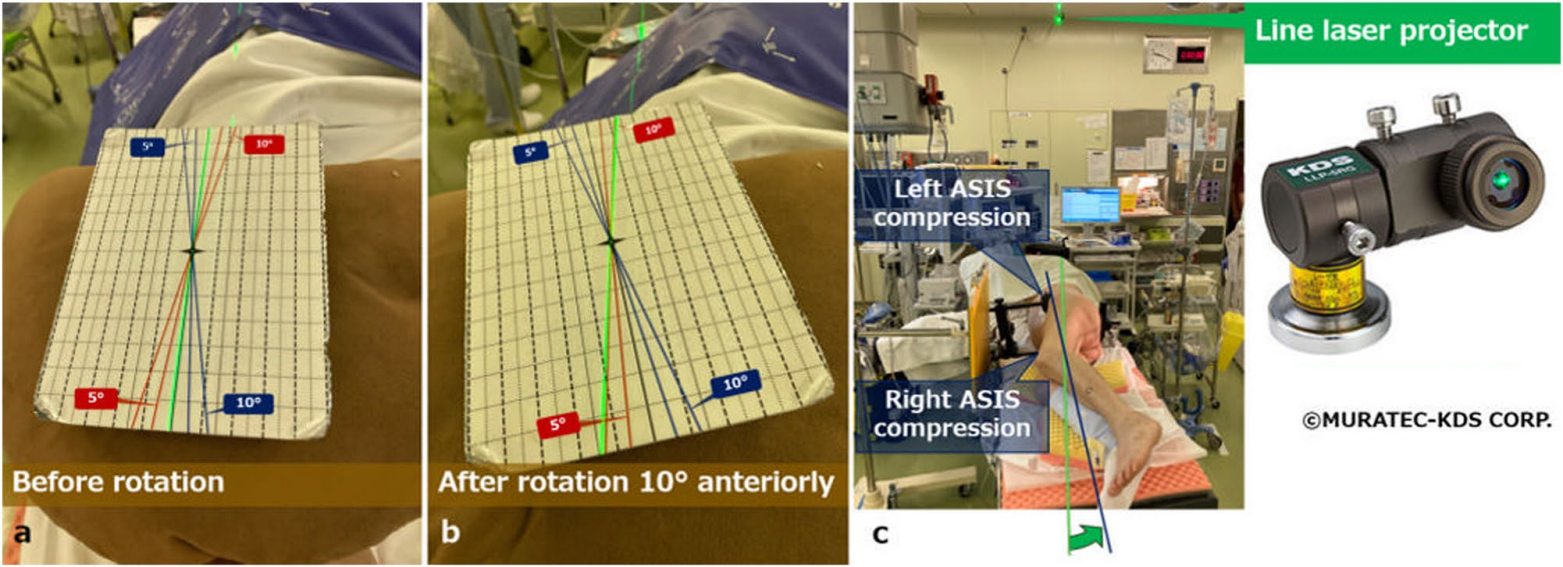
(**a**) Before operating table rotation. The patient is in the right lateral decubitus position. (**b**) The operating table was rotated 10° anteriorly, in this case, to adjust the pelvis tilt. A green laser beam can serve as an angular guide. (**c**) The pelvic tilt was adjusted by rotating the operating table guided by a green laser beam. A line laser projector is fixed on the ceiling of the operating room. Our pelvic lateral positioner can control the compression of the left or right anterior superior iliac spine (ASIS) independently to adjust the anterior or posterior pelvic rotation.

1. Conventional THA (April 2010–November 2011): A cup was placed in reference to acetabular anatomical landmark using an operative angle cup alignment guide (operative inclination 45° and operative anteversion 20°) though target cup angles were RI 45° and RA 20°.
2. THA with laser guide technique (December 2011–October 2012): Since December 2011, a laser guide technique for preoperative postural adjustment to the FPP in the lateral decubitus position and intraoperative angular reference was introduced. Specifically, an anteroposterior (AP) radiograph of the pelvis with a patient in the lateral decubitus position was taken just before surgery in the operating room. The radiograph was compared with prepared several patterns of pelvic DRRs (Fig. 2) and then the pelvis was adjusted to FPP orientation^24^. First, the pelvic tilt was adjusted by rotating the operating table with a laser beam as an angular guide (Fig. 3a,b), which was emitted from a line laser projector (LLP-5RG, Muratec-KDS, Kyoto, Japan) fixed on the ceiling of the operating room (Fig. 3c). Second, pelvic rotation was adjusted by handling the compression of the anterior superior iliac spines by a pelvic lateral positioner (Fig. 3c). Another AP pelvic radiograph was taken to evaluate the correction. Unless the pelvis was FPP, repeated the procedure to obtain FPP. Finally, pelvic misalignment in the coronal plane was adjusted by tilting the operating table cranially or caudally with a digital protractor (Fig. 4a). This tilting angle was measured as an angle formed by a perpendicular line to the floor (a hanging chain) and a teardrop line (Fig. 4b). An operative angle cup alignment guide was still used, even though target cup angles were RI 40° and RA 20°.

**Figure 4:**
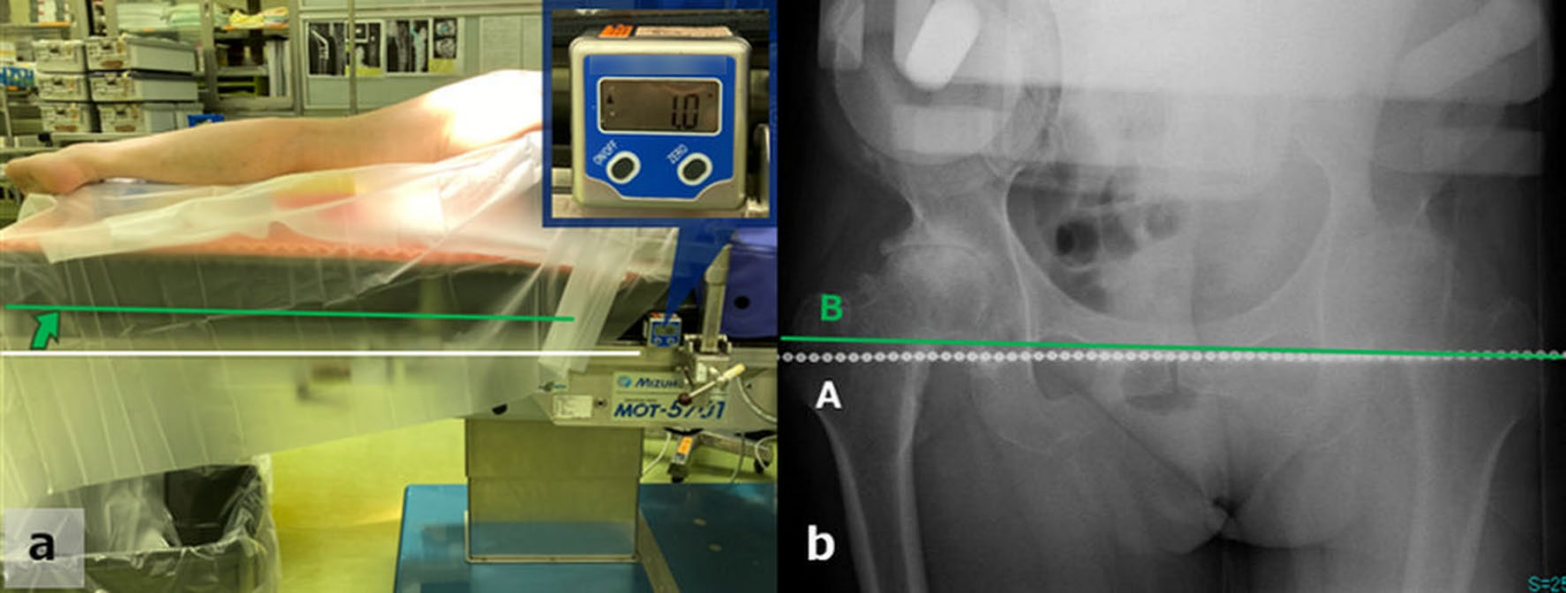
(**a**) The operating table was tilted 1° cranially, in this case, to adjust the pelvis to the functional pelvic plane. (**b**) This tilting angle (pelvic tilt in the coronal plane) is measured as an angle formed by a perpendicular line to the floor (a hanging chain, line A) and a teardrop line (line B).
3. THA with laser guide technique using a radiographic cup alignment guide (November 2012–March 2016): Since November 2012, a radiographic angle cup alignment guide (RI 40° and RA 20°) has been introduced. When a laser beam and this alignment guide are parallel and a level gauge indicates 0° in both the coronal and axial planes, the cup is supposed to be in RI 40° and RA 20° (Fig. 5).

**Figure 5:**
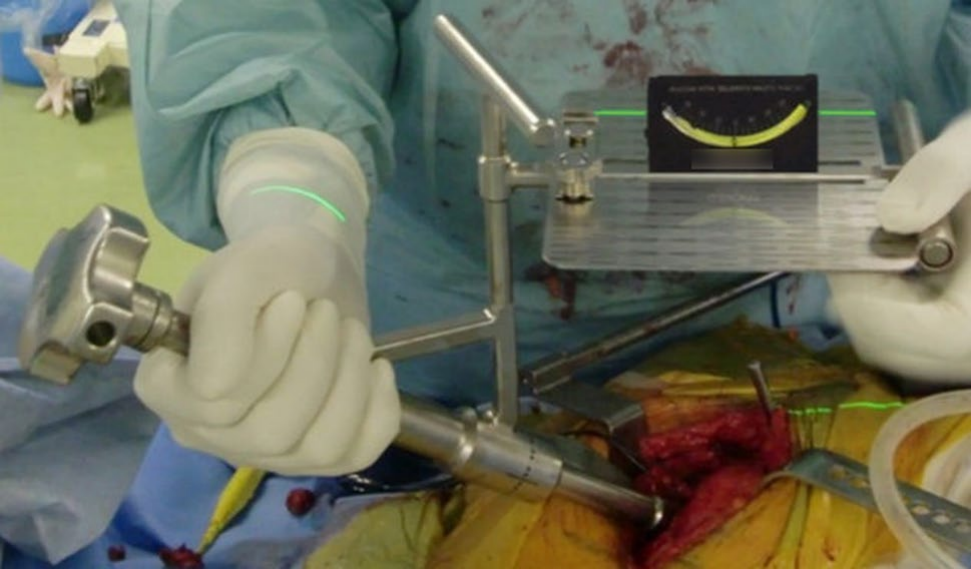
When a green laser beam and a radiographic alignment guide are parallel, and a level gauge indicates 0° in both coronal and axial planes, a cup is supposed to be in 40° of radiographic inclination and 20° of radiographic anteversion.

### Postoperative radiographic measurement of RI and RA and assessment of accuracy

**Figure 6:**
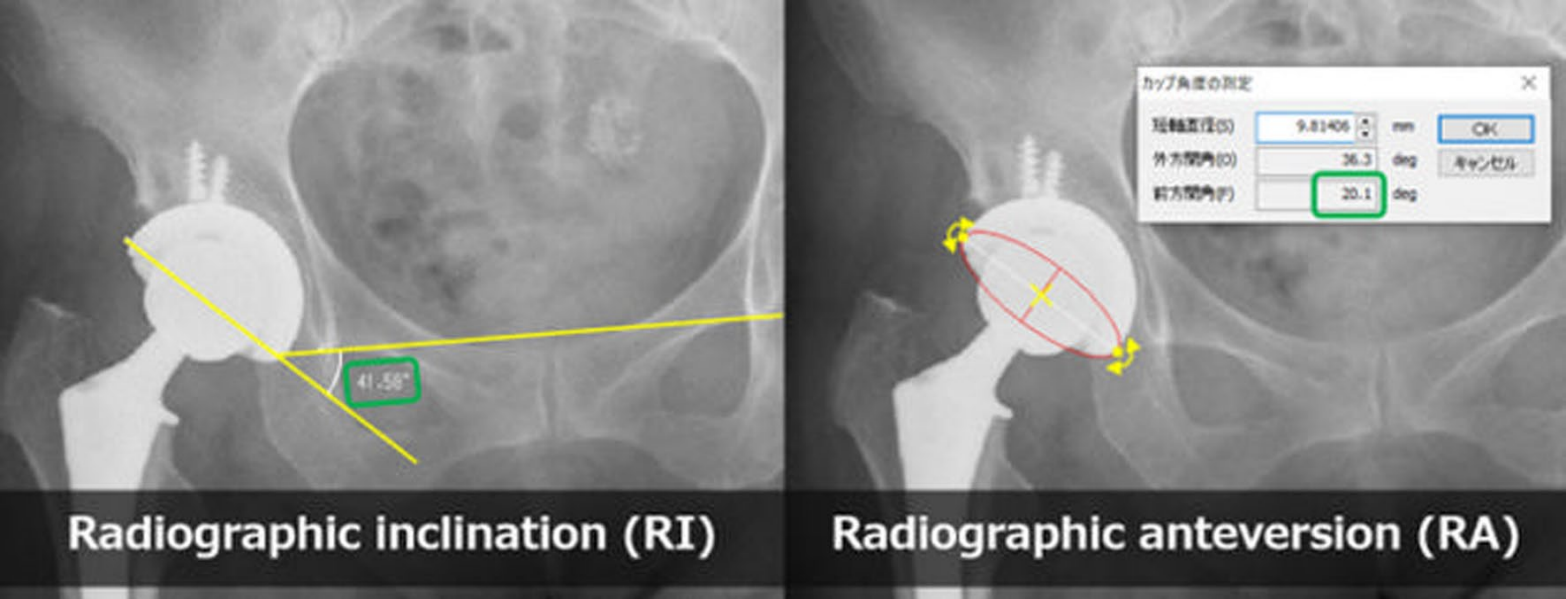
Postoperative radiographic inclination and radiographic anteversion were measured using 2D Template (Kyocera Medical).

Postoperative RI and RA were measured using 2D Template (Kyocera Medical) (Fig. 6). To assess accuracy, the difference between the target angle and actual angle was calculated in RI and RA, respectively. The percentage of cases of both RI and RA errors within 5° and 10° was also reported. Measurements were repeated at least one month after the initial measurements by two reviewers (YK and TN). The intraobserver reliability of radiographic measurement was almost perfect according to the intraclass correlation coefficient (0.98 (95% CI 0.96–0.99) for RI and 0.97 (95% CI 0.95–0.98) for RA). The interobserver reliability was also almost perfect (0.94 (95% CI 0.75–0.98) for RI and 0.91 (95% CI 0.84–0.95) for RA)^25^.

### Postoperative precautions and complications including dislocation

Precautions against postoperative dislocation of THA with posterolateral approach included some prohibited hip positions and movements. The patients were educated through the rehabilitation program and booklet. As a clinical outcome, dislocation rate was mainly reviewed in the patients who were followed up for > 2 years postoperatively. The patients who were lost to follow-up in < 2 years were also reviewed, but excluded them from the analysis. In addition, complications other than dislocation were also reviewed.

### Statistical analysis

Descriptive statistics are presented as the number and percentage or as the mean and standard deviation (SD). The Steel–Dwass test was used to compare the measured angles among the conventional, laser guide, and laser + radiographic alignment guide groups, and Pearson’s chi-square test was used to compare categorical variables among the three groups. The Levene’s test was used to assess the equality of variances for RI and RA between the conventional group and the laser groups (laser guide group and laser + radiographic alignment guide group). The Wilcoxon rank-sum test was used to compare continuous variables and the Fisher’s exact test was used to compare categorical variables between the dislocation and no dislocation groups. Statistical analysis was performed using JMP Pro 14.2.0 (SAS Institute Inc., Cary, NC, USA). Each p value < 0.05 was considered statistically significant.

## Results

**Figure 7:**
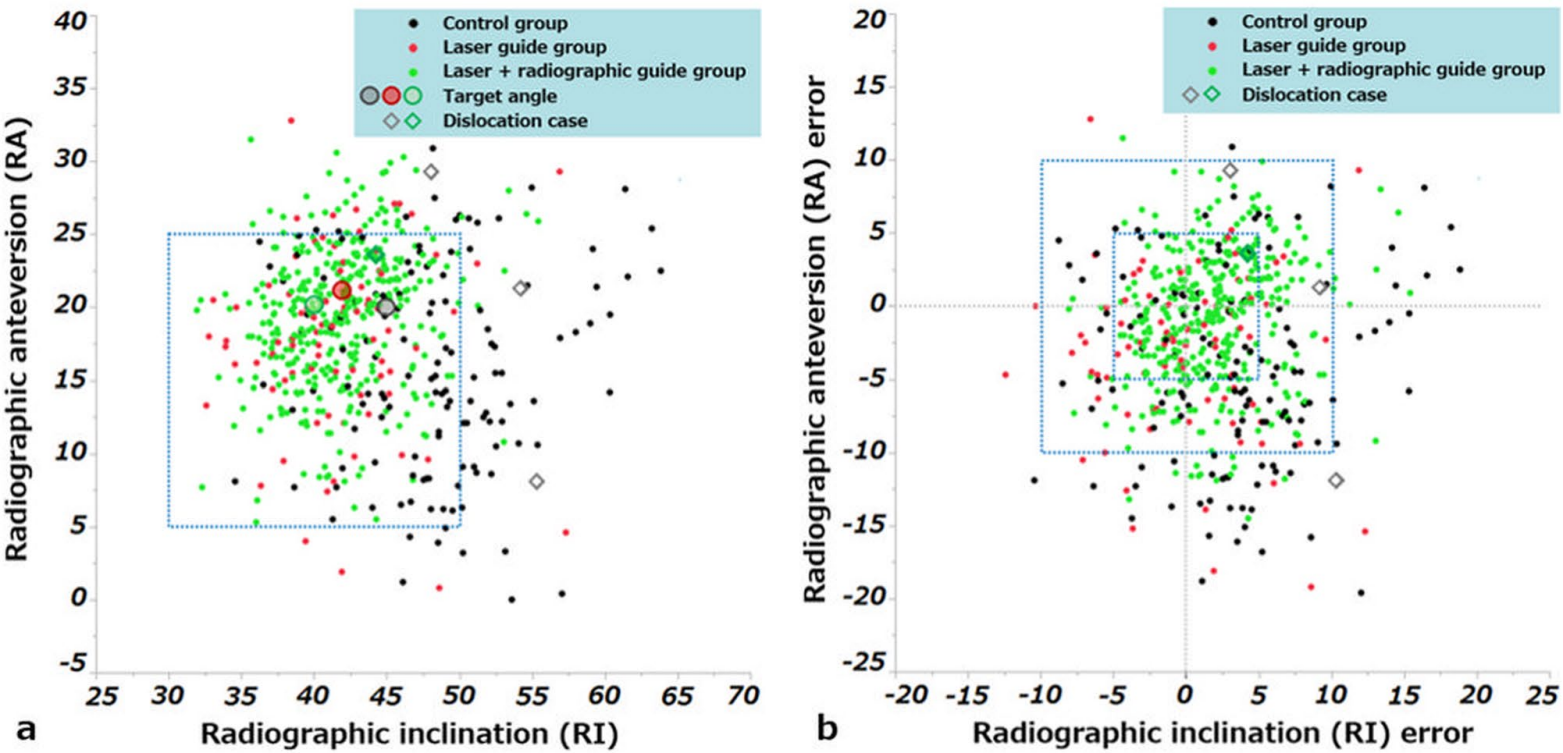
(**a**) Scatter diagram of postoperative radiographic inclination (RI) and radiographic anteversion (RA). Average target angle in each group and dislocation cases are indicated in other shapes. The Lewinnek safe zone (cup inclination 40° ± 10° and anteversion 15° ± 10°)^26^ is included for reference. (**b**) Scatter diagram of the RI and RA errors. Dislocation cases are indicated in another shape. The auxiliary square indicates both RI and RA errors within 5° and 10°.

The mean target angles of RI and RA in the conventional, laser guide, and laser + radiographic alignment guide groups were 45.0° ± 0.4° and 20.1° ± 2.0°, 41.8° ± 2.6° and 21.2° ± 2.0°, and 40.0° ± 0.2° and 20.3° ± 1.8°, respectively. Actual angles of RI and RA in the three groups were 48.3° ± 5.8° and 15.9° ± 7.0°, 41.7° ± 4.7° and 18.0° ± 6.2°, and 41.9° ± 3.7° and 19.5° ± 4.7°, respectively (Table 1). Error angles of RI and RA in the three groups were 3.3° ± 5.8° and − 4.2° ± 6.7°, − 0.1° ± 4.9° and − 3.2° ± 5.7°, and 1.9° ± 3.7° and − 0.8° ± 4.5°, respectively. Scatter diagrams of actual RI and RA, with the Lewinnek safe zone^26^ for reference, are shown in Fig. 7a. Absolute values of the RI and RA error in the three groups were 5.3° ± 4.0° and 6.5° ± 4.5°, 4.0° ± 2.8° and 4.9° ± 4.4°, and 3.3° ± 2.6° and 3.6° ± 2.8°, respectively (Table 2). The absolute value of RI error in the laser + radiographic alignment guide group was significantly smaller than that in the laser guide and conventional groups (p = 0.044 and p < 0.001, respectively). The laser guide group showed a trend of small error than that in the conventional group (p = 0.077). Absolute values of RA error in the laser guide and laser + radiographic alignment guide groups were significantly smaller than that in the conventional group (p = 0.014 and p < 0.001, respectively). The percentage of the error within 5° or 10° is shown in Table 3, indicating a significant improvement of accuracy with the introduction of the laser guide technique and a radiographic cup alignment guide. The cases of both RI and RA errors within 5° in the conventional, laser guide, and laser + radiographic alignment guide groups were 24% (32/135 hips), 49% (39/80 hips), and 62% (237/384 hips), respectively. Those within 10° in the three groups were 67% (91/135 hips), 84% (67/80 hips), and 95% (363/384 hips), respectively. A scatter diagram of RI and RA errors was shown in Fig. 7b.

**Table 1:** Target, actual, and error angles of RI and RA.

	Conventional group (n = 135)	Laser guide group (n = 80)	Laser + radiographic alignment guide group (n = 384)
Target RI (°)	45.0 ± 0.4	41.8 ± 2.6	40.0 ± 0.2
Target RA (°)	20.1 ± 2.0	21.2 ± 2.0	20.3 ± 1.8
Actual RI (°)	48.3 ± 5.8	41.7 ± 4.7	41.9 ± 3.7
Actual RA (°)	15.9 ± 7.0	18.0 ± 6.2	19.5 ± 4.7
RI error (°)	3.3 ± 5.8	− 0.1 ± 4.9	1.9 ± 3.7
RA error (°)	− 4.2 ± 6.7	− 3.2 ± 5.7	− 0.8 ± 4.5

Values are expressed as mean with standard deviation. *RI* radiographic inclination, *RA* radiographic anteversion.

**Table 2:** Absolute values of RI and RA errors.

	Conventional group (n = 135)	Laser guide group (n = 80)	Laser + radiographic alignment guide group (n = 384)
Absolute value of RI error (°)	5.3 ± 4.0	4.0 ± 2.8	3.3 ± 2.6*^,†^
Absolute value of RA error (°)	6.5 ± 4.5	4.9 ± 4.4*	3.6 ± 2.8*

Values are expressed as mean with standard deviation. *Significant compared with the conventional group. ^†^Significant compared with laser guide group. *RI* radiographic inclination, *RA* radiographic anteversion.

**Table 3:** Percentage of the error less than 5° and 10° in both RI and RA.

	Conventional group (n = 135)	Laser guide group (n = 80)	Laser + radiographic alignment guide group (n = 384)	p value
RI and RA < 5°	24% (32 hips)	49% (39 hips)	62% (237 hips)	< 0.001
RI and RA < 10°	67% (91 hips)	84% (67 hips)	95% (363 hips)	< 0.001

*RI* radiographic inclination, *RA* radiographic anteversion.

A total of 540 hips in 476 patients were followed up > 2 years postoperatively and the average follow-up period was 5.2 years (range, 2.0–10.8 years). In the other 59 hips in 55 patients, there was no early-term dislocation^27^ within their < 2 years follow-up period. The dislocation rate in the conventional, laser guide, and laser + radiographic alignment guide groups were 2.5% (3/119 hips), 0% (0/78 hips), and 0.3% (1/343 hips), respectively. The dislocation rate in the laser groups (0.2%, 1/421 hips) was significantly lower than that in the conventional group (p = 0.035). The conventional and laser groups exhibited significant variances in both RI (SD were 5.8° and 3.9°, respectively, p < 0.001) and RA (SD were 7.0° and 5.0°, respectively, p < 0.001), meaning that cup aligment variations were reduced by the laser guide technique. There was no significant difference in the patients’ demographics and femoral head size between the groups (Table 4).

**Table 4:** Demographic data and surgical information.

	Dislocation group (n = 4)	No dislocation group (n = 536)	p value
Age (years)	68.5 ± 2.9	68.0 ± 9.4	0.92
Sex (male/female)	1/3	104/431	0.58
BMI (kg/m^2^)	25.4 ± 3.2	24.1 ± 4.3	0.55
Diagnosis (OA/ONFH/FNF/RA/others)	3/1/0/0/0	431/27/28/16/34	0.45
Femoral head size (< 28 mm/> 32 mm)	1/3	250/286	0.63
Actual RI (°)	50.4 ± 5.2	43.3 ± 5.1	< 0.001
Actual RA (°)	20.6 ± 9.0	18.7 ± 5.6	0.50
Conventional/Laser	3/1	116/420	0.035

Values are expressed as mean with standard deviation. *BMI* body mass index, *OA* osteoarthritis, *ONFH* osteonecrosis of the femoral head, *FNF* femoral neck fracture, *RA* rheumatoid arthritis, *RI* radiographic inclination, *RA* radiographic anteversion.

Complications other than dislocation were as follows: Intraoperative femoral fracture, which required additional wiring (6 hips); deep infection, which required surgical intervention (5 hips); and postoperative periprosthetic fracture, which required open reduction and internal fixation (2 hips). There was no postoperative sciatic or femoral nerve palsy, symptomatic pulmonary embolism, and aseptic loosening.

## Discussion

In modern THA, three-dimensional preoperative planning is being common because it is helpful to define the optimal component size, position, and orientation^28^. Accurate implant alignment accordingly leads to reduced intra- and postoperative complications and may lead to better implant longevity. We developed a novel laser guide technique for accurate cup alignment and introduced the technique here. Although the difference may be small, our laser guide technique substantially enhanced the cup alignment accuracy compared with a conventional manual technique. The dislocation rate using the conventional technique was 2.5%, whereas that using the laser guide technique was 0.2%.

The reported inaccuracy of manual cup placement in THA with posterolateral approach was 6.1°–6.5° for inclination and 5.5°–9.0° for anteversion^17,29^, which was similar to our conventional THA (5.3° for inclination and 6.5° for anteversion). Wide postural variation in surgery with patients in the lateral decubitus position potentially contributed to this inaccuracy. In a previous study, when the patients were placed in the lateral decubitus position before surgery, an error of > 5° from the FPP were as follows: 43% in the sagittal plane, 47% in the axial plane, and 12% in the coronal plane^19^. That study suggested that preoperative positional adjustment would thus be a crucial step for accurate cup alignment when the navigation system is unavailable. In the present study, it was demonstrated that the laser beam served as an angular navigation in the preoperative postural adjustment and intraoperative cup alignment, and thus, the accuracy of cup alignment markedly increased (3.3° for inclination and 3.6° for anteversion). On the contrary, the reported error of the cup angle using CT-based navigation in THA with posterolateral approach was 1.4°–1.8° for inclination and 1.2°–2.7° for anteversion^14,15^. If the high cost was disregarded, better ways to place a cup accurately would be CT-based navigation or a robotic arm-assisted system^30^. Although our laser guide technique may be less accurate compared with these expensive systems, we believe it is also a good option in that it is cost-effective (the line laser projector costs approximately 40,000 JPY (350 USD) and the running cost is not required), easy for any hospital to introduce, and relatively accurate in manual cup placement methods.

In a systematic review, the dislocation rates for the posterior approach THA with capsular repair was 1.01–2.03%^7,9^. Compared with these reports, our dislocation rate in posterolateral THA with laser guide technique was even lower. The reduced variation and error of cup alignment using the laser guide technique might contribute to the lower dislocation rate, yet ideal implant alignment does not always prevent dislocation. A patient who dislocated in the laser + radiographic guide group has placed the cup in RI of 44° and RA of 23°, both within 5° errors (Fig. 7), and stem anteversion was 40°. The implant alignment seemed to be in an acceptable range and offset and leg length were also appropriate; however, the surgery was performed because of alcohol-induced osteonecrosis of the femoral head. In addition, the patient was still in a state of inebriation habitually, which led to a single posterior dislocation one month postoperatively. The cause of dislocation after THA is well known as multifactorial^31,32^; however, accurate cup placement should be pursued because implant alignment is considered of primary importance for the long-term THA survival^32^.

Several limitations of this study should be acknowledged. First, although true FPP in the lateral decubitus position is obtained before surgery, the pelvis may move during surgery^19^. A weakness of our technique is that it cannot track pelvic movement during surgery like the navigation system. As a practical measure, an intraoperative control radiograph after cup placement was taken not to overlook unexpected implant malposition. Preoperative positional adjustment also makes the intraopereative radiographic evaluation easier because of less pelvic misalignment. Second, because clinical data or patient satisfaction scores were unavailable in most of the initial patients, only the dislocation rate was reviewed as a clinical outcome. Additionally, this is not a randomized controlled study. There is an unmeasured confounder, such as stem anteversion or offset, but at least, there was no difference between the groups with or without dislocation in reviewed patient demographics and femoral head size. This study does not directly provide an association between the laser guide technique (accurate cup alignment) and the reduced dislocation rate; however, it does present several evidence that this relationship exists. It has been reported that accurate cup positioning by CT-based navigation was associated with lower dislocation rate and lower impingement-related mechanical complications in THA over a minimum 10-year follow-up^32^, which suggests that our laser guide technique may also be clinically beneficial.

In conclusion, our laser guide technique: a novel method for accurate acetabular cup alignment in THA significantly decreased cup alignment error and variation compared to the conventional manual technique, suggesting its potential applicability in clinical practice. Our dislocation rate was 0.2% in THA with the piriformis-sparing posterolateral approach using this novel technique. Although it may be less accurate compared with CT-based navigation system, this cost-effective technique is also a useful option for THA in the lateral decubitus position.

## Data Availability

The datasets generated during and/or analyzed during the current study are available from the corresponding author on reasonable request.
